# Metallic Strip Gratings in the Sub-Subwavelength Regime

**DOI:** 10.3390/s140711786

**Published:** 2014-07-04

**Authors:** Adriana Savin, Rozina Steigmann, Alina Bruma

**Affiliations:** 1 Nondestructive Testing Department, National Institute of R&D for Technical Physics, 47 D., Mangeron Blvd, 700050 Iasi, Romania; E-Mail: steigmann@phys-iasi.ro; 2 Faculty of Physics, Al.I.Cuza University, 11 Carol I Blvd, 700506 Iasi, Romania; 3 CRISMAT Laboratory, National Graduate School of Engineering, University of Caen on Normandy, 6 Marechal Juin Blvd, Caen 14050, France; E-Mail: bruma.alina@ensicaen.fr

**Keywords:** sub-subwavelength regime, sensors with metamaterials, metallic strip grating, evanescent modes

## Abstract

Metallic strip gratings (MSG) have different applications, ranging from printed circuits to filters in microwave domains. When they are under the influence of an electromagnetic field, evanescent and/or abnormal modes appear in the region between the traces, their utilization leading to the development of new electromagnetic nondestructive evaluation methods. This paper studies the behavior of MSGs in the sub-subwavelength regime when they are excited with TE_z_ or TM_z_ polarized plane waves and the slits are filled with different dielectrics. The appearance of propagating, evanescent and abnormal modes is emphasized using an electromagnetic sensor with metamaterials lens realized with two conical Swiss rolls, which allows the extraction of the information carried by the guided evanescent waves. The evanescent waves, manipulated by the electromagnetic sensor with metamaterial lenses, improve the electromagnetic images so that a better spatial resolution is obtained, exceeding the limit imposed by diffraction. Their theoretical and experimental confirmation opens the perspective for development of new types of sensors working in radio and microwave frequencies.

## 1. Introduction

The metal strip grating is a periodic planar arrangement of parallel metal strips with infinite length and infinitesimal thickness. Metallic strip gratings have specific applications as filters or conductive strips or micro-strips, traces in rigid or flexible printed circuits. The interaction of electromagnetic fields with periodical metallic structures is interesting from both fundamental as well as practical points of view [1–4]. In subwavelength optics, the usual Huygens principle-based approaches fail [5], and the solutions for electromagnetic diffractions and transmission enhancement of electromagnetic waves by means of subwavelength metallic apertures are relatively complicated. Lord Rayleigh has advanced an approach to diffraction calculation in his solution to wave scattering from a reflecting grating [6] when the size or periodicity of diffracting object becomes comparable to or smaller than the wavelength of the incident electromagnetic wave. By expressing the wave as a superposition of plane-wave harmonics, he obtained the diffraction amplitudes through boundary conditions fitting at the grating surface. This yields to reasonable results for shallow gratings even when the periodicity is smaller than *λ*. However, when the ratio of grating depth to periodicity exceeds a small critical value, the method fails to converge [7]. A direct numerical solution of the wave equation in differential form is reported. This shows instabilities for good conductor strips, such as aluminium or silver [1]. The integral equation approach, on the other hand, is numerically more stable than the differential method but can display matrix ill-conditioning problems when grating depth becomes too large [1].

It became clear that the basic concept of the eigenmodes in wave-guiding structures, which qualifies the modes into propagating and evanescent, is not fully applicable to metal-based structures [8,9]. The case in which the incident field at metallic strip grating is TM_z_ polarized [9–13] was intensively studied since the excitation of surface plasmon polaritons is possible [14,15]. The behavior of a metallic strip grating when the incident field is TE_z_ polarized has been less studied [16].

In all these approaches, it is presumed that the dielectric from the slits is the air (with the electromagnetic properties of the vacuum *ε_0_*, *μ_0_*), the metallic strip grating constant being smaller than the wavelength of the incident electromagnetic field. This case is defined as sub-wavelength regime. When the incident electromagnetic wave is a plane wave, TM_z_ polarized; it has been demonstrated that a propagating mode and more evanescent and abnormal modes are generated in slits [9,17,18]. As specified in reference [19], when the constant of a metallic strip silver grating is 1 mm and the incident wave is TM_z_ polarized, having a 0.6 m wavelength, in it has been shown that only a single evanescent mode appears in slits; this mode disappears when water is inserted in slits (*ε*_water_ = 81) [20].

This paper proposes a theoretical and experimental study of the eigenmodes that appear in a metallic strip grating having thick metallic strips from silver, operating in sub-subwavelength regime; this implies the constant of strip grating much smaller than the wavelength of incident electromagnetic field. This approach is new for stratified structures (MSG, dielectrics and metamaterials) in subwavelength regime and can be integrated into a new class of sensors with performances able to open new domains of applications in electromagnetic evaluation of composite and nanocomposite materials.

Both TE_z_ and TM_z_ polarized plane waves are used as incident to metallic strip grating. The “visualization” of eigenmodes is experimentally made using a transducer with metamaterials lens, when dielectric fluids with different dielectric constants are inserted in slits. These lenses allow the manipulation and focusing of evanescent waves that appears in the slits, leading to the spectacular improvement of the spatial resolution, superior to those described in [19]. They offer the possibility to ameliorate the quality of the electromagnetic images using the evanescent waves [5] that appears in slits and their transfer at distance. The composite materials, CFRP type, due to carbon fibers conductivity, can be considered as strip grating conductive stratified structures. Nowadays, these composites have large usage and they require electromagnetic nondestructive evaluation methods with high sensitivity in order to detect moisture presence, fibers breaking, delaminations due low energy impacts, *etc.* A good agreement between theoretical and the experimental results is found.

## 2. Basic Model

**Figure 1.**
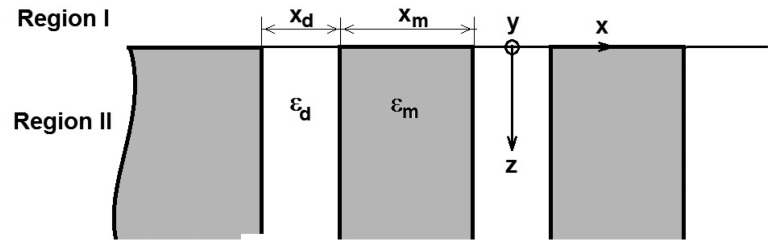
Semi-infinite thick metallic strip grating.

Considering the semi-infinite periodic grating of metallic strips and slits filled with the dielectric as shown in Figure 1, the space above the grating (Region I) is assumed to be vacuum. The dielectric permittivity of the metal is ε_m_ and of the dielectric in the slits is ε_d_. If the thickness of the metal strips, *h*, is at least three times the standard penetration depth of the incident electromagnetic field, the strip grating can be considered thick:

(1a) h>3δ

where:

(1b) $$\delta =\sqrt{\frac{2}{\omega \mu \sigma }}$$

*ω* is the angular speed of the incident electromagnetic field, *σ* and *μ* are the electrical conductivity and the magnetic permeability, respectively, of the metallic strips.

In this case, the metallic strip grating can be considered occupying the entire semi-plane z < 0 (Region II in Figure 1).

For a metallic strip grating excited with a plane wave TEz polarized, the electric field of the incident plane wave is parallel to the y axis, so that *E_x_* = *E_z_* = 0 and *E_y_* ≠ 0.

In the case of TM_z_ polarization, the magnetic field of the incident plane wave is parallel to the *y* axis, so that *H_x_* = *H_z_* = 0 and *H_y_* ≠ 0. For both polarizations, normal incidence will be taken into consideration.

In Region I, the electromagnetic field can be expressed as a Fourier series:

(2) $$\Psi_{I}=\Psi_{0}\exp\left(ik_{0}z\right)+\sum_{n=0}^{\infty }a_{n}\cos\left(\text{nKx}\right)\exp\left(-K_{n}z\right)$$

where Ψ*_I_* is the y component of the electric/magnetic field for the TE_z_/TM_z_ polarized wave, Ψ_0_ is the amplitude of the electric/magnetic incident field, *a_n_* represents the amplitude of the scattered electric/magnetic field component, $k_{0}=\frac{2\pi }{\lambda }=\frac{\omega }{c}$, $K=\frac{2\pi }{x_{0}}$, *x*_0_ = *x_d_* + *x_m_* represents the strip grating constant, $i=\sqrt{-1}$ and $K_{n}=\sqrt{n^{2}K^{2}-k_{0}^{2}}$. The other components of the electromagnetic field can be easily calculated using the Maxwell's equations.

In Region II, the field in the grating can be expressed as an expansion in the eigenmodes of the periodic structure:

(3) $$\psi_{\text{II}}=\sum_{v=1}^{\infty }b_{v}\theta_{v}\left(x\right)\exp\left(i\beta_{v}z\right)$$

where *ψ_II_* is the y component of the electric/magnetic field in the period strips, *b_v_* is the amplitude of *v* eigenmodes, *θ_v_* (*x*) is the y component of the electric/magnetic eigenmodes and *β_v_* is the propagation constant with positive imaginary part for *v* eigenmodes:

(4) $$\theta_{v}\left(x\right)=\{\begin{array}{lll} e_{v}\left(x\right),\text{for} & {\text{TE}}_{\text{z}} & \text{polarized incident electromagnetic field}\\ h_{v}\left(x\right),\text{for} & {\text{TM}}_{\text{z}} & \text{polarized incident electromagnetic field} \end{array}$$

Using [7], the eigenmodes can be expressed as:

(5) $$e_{v}\left(x\right)=\{\begin{array}{ll} \sin\left(p_{m}\frac{x_{m}}{2}\right)\sin\left(p_{d}x\right), & -\frac{x_{d}}{2}\leq x\leq \frac{x_{d}}{2}\\ \sin\left(p_{d}\frac{x_{d}}{2}\right)\sin\left(p_{m}\left(x-\frac{x_{0}}{2}\right)\right), & \left(-\frac{x_{0}}{2}\leq x\leq -\frac{x_{d}}{2}\right)\cup \left(\frac{x_{d}}{2}\leq x\leq \frac{x_{0}}{2}\right) \end{array}$$

(6) $$h_{v}\left(x\right)=\{\begin{array}{ll} \cos\left(p_{m}\frac{x_{m}}{2}\right)\cos\left(p_{d}x\right), & -\frac{x_{d}}{2}\leq x\leq \frac{x_{d}}{2}\\ \cos\left(p_{d}\frac{x_{d}}{2}\right)\cos\left(p_{m}\left(x-\frac{x_{0}}{2}\right)\right), & \left(-\frac{x_{0}}{2}\leq x\leq -\frac{x_{d}}{2}\right)\cup \left(\frac{x_{d}}{2}\leq x\leq \frac{x_{0}}{2}\right) \end{array}$$

where:

(7) $$\begin{array}{c} p_{m}=\sqrt{\varepsilon_{m}k_{0}^{2}-\beta_{v}^{2}}\\ p_{d}=\sqrt{\varepsilon_{d}k_{0}^{2}-\beta_{v}^{2}} \end{array}$$

## 3. Eigenmodes and Dispersion Equation

### 3.1. Theoretical Development

The metals used in fabrication of metallic strip grating (especially silver) have complex dielectric permittivity $\varepsilon_{m}=\varepsilon_{m}^{\prime }+i\varepsilon_{m}^{\prime \prime }$ with large negative real part $\varepsilon_{m}^{\prime }$ and a relatively small imaginary part $\varepsilon_{m}^{\prime \prime }$ [21].

Using the continuity of the tangential field components together with the Bloch condition for the periodic structure, we can obtain the dispersion equation, which allows the determination of the eigenvalue *β_v_*. For the excitation of metallic strip grating with an electromagnetic planar wave TE_z_ polarized, the dispersion equation becomes:

(8) $$\frac{1}{2}\left(\frac{p_{d}}{p_{m}}+\frac{p_{m}}{p_{d}}\right)\sin\left(p_{m}x_{m}\right)\sin\left(p_{d}x_{d}\right)=\cos\left(p_{m}x_{m}\right)\cos\left(p_{d}x_{d}\right)-1$$

and for excitation of metallic strip grating with an electromagnetic planar wave TM_z_ polarized, the dispersion equation becomes:

(9) $$\frac{1}{2}\left(\frac{p_{d}\varepsilon_{m}}{p_{m}\varepsilon_{d}}+\frac{p_{m}\varepsilon_{d}}{p_{d}\varepsilon_{m}}\right)\sin\left(p_{m}x_{m}\right)\sin\left(p_{d}x_{d}\right)=\cos\left(p_{m}x_{m}\right)\cos\left(p_{d}x_{d}\right)-1$$

The dispersion Equations (8) and (9) are transcendental complex and can be only numerically solved. These equations provide the eigenvalues—the propagation constants *β_v_* of different modes that are generated in slits for various excitations.

The analytical solution of Maxwell's equations in each of the two spatial regions (see Figure 1) allows to expand arbitrarily the wave field *Ψ*, as a superposition of eigenfunctions. The expansion coefficients through the boundary conditions at z = 0 can be calculated:

(10) $$\begin{array}{c} \psi^{I}\left(x,z=0\right)=\psi^{II}\left(x,z=0\right)\\ \frac{\partial \psi^{I}}{\partial z}\left(x,z=0\right)=\frac{\partial \phi^{II}}{\partial z}\left(x,z=0\right) \end{array}$$

where *ϕ^II^* is defined as:

(11) $$\phi^{\text{II}}=\{\begin{array}{llll} \overset{\infty }{\underset{v=1}{\text{∑}}}b_{v}e_{v}\left(x\right)\exp\left(i\beta_{v}z\right), & \text{for} & {\text{TE}}_{\text{z}} & \text{polarization}\\ \overset{\infty }{\underset{v=1}{\text{∑}}}\frac{1}{\varepsilon \left(x\right)}b_{v}h_{v}\left(x\right)\exp\left(i\beta_{v}z\right) & \text{for} & {\text{TM}}_{\text{z}} & \text{polarization} \end{array}$$

Applying the Fourier transform to the continuity Equation (10), the system of algebraic equations for the amplitudes of the field harmonics can be easily obtained.

For the excitation of metallic strip grating with an electromagnetic planar wave TE_z_ polarized, Equations (5), (10) and (11) lead to the algebraic system of equations:

(12) $$\begin{array}{l} \psi_{0}\delta_{n_{0}}+a_{n}=b_{v}<e_{v}{>}_{n}\\ ik_{0}\psi_{0}\delta_{n_{0}}-iK_{n}a_{n}=ib_{v}<e_{v}{>}_{n} \end{array}$$

and:

(13) $${\langle e_{v}\rangle }_{n}=\int_{\begin{array}{cc} \text{one} & \text{period} \end{array}}e_{v}\left(x\right)\cos\left(\text{nKx}\right)dx$$

where δ is the Kronecker symbol.

For the excitation of the metallic strip grating with an electromagnetic planar wave TM_z_ polarized, Equations (6), (10) and (11) lead to:

(14) $$\begin{array}{l} \psi_{0}\delta_{n_{0}}+a_{n}=b_{v}<e_{v}{>}_{n}\\ ik_{0}\psi_{0}-iK_{n}a_{n}=ib_{v}\beta_{v}<\frac{h_{v}}{\varepsilon }{>}_{n} \end{array}$$

where:

(15) $$\langle \frac{h_{v}}{\varepsilon }\rangle =\int_{-\frac{x_{d}}{2}}^{\frac{x_{d}}{2}}\frac{h_{v}\left(x\right)}{\varepsilon_{d}}dx+\int_{\frac{x_{d}}{2}}^{\frac{x_{d}}{2}+x_{m}}\frac{h_{v}\left(x\right)}{\varepsilon_{m}}dx$$

### 3.2. Numerical Results

The case of a metallic strip grating made from silver strips having 10 μm thickness, the width of strips being *x_m_* = 0.6 mm and the width of slits being x_d_ = 0.4 mm is analyzed, considering that the wavelength of the incident field is *λ* = 0.6 m (corresponding to 500 MHz frequency).

The electrical conductivity of silver is *σ_Ag_* = 6.2873 × 10^7^ S/m [22] such that the metallic strip grating is fulfilling the condition given by Equation 1a, as being infinitely thick. According to reference [22], at frequencies around 500 MHz, the dielectric permittivity of silver is *ε_m_* =−48.8+*i*·3.16.

Considering that the slits are filled with different liquid dielectrics, having the dielectric constant ε_d_ indicated in Table 1 [23], the solutions of dispersion Equations (8) and (9) are quest having the form:

(16) $$\beta =\beta^{\prime }+i\beta^{\prime \prime }$$

where *β″* > 0. The obtained results are presented in Table 1.

Table 1 shows that for metallic strip grating excitation with electromagnetic planar waves TE_z_ polarized, only one evanescent mode is generated in slits. The imaginary component of the propagation constant of these modes is increasing with the increase of dielectric constant of the liquid inserted in slits.

For metallic strip grating excitation with electromagnetic planar waves TM_z_ polarized, at relatively small values of dielectric constants, only evanescent modes are generated in slits, and when *ε_d_* ≥ 4.81 , the case of chloroform, abnormal modes are generated in slits. Equation (9) admits pairs of solutions (there are abnormal modes) *β_v_* = *β′* +*iβ″* and respectively *β_v_* = *β′* +*iβ″*, that correspond to a modification of the phase in advance or retarded with same value, therefore these modes are equivalent due to the periodicity conditions. Knowing the eigenvalues for the propagation constant in the cases of both polarizations of the incident field, the form of eigenmodes can be calculated using Equations (5)–(7) and the data from Table 1.

**Figure 2.**
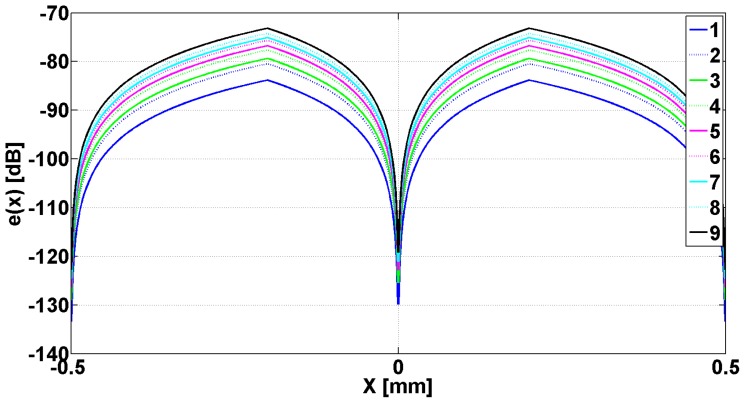
Eigenmodes *e*(*x*) plotted as a function of *x* for one period of metallic strip grating. Numbers in legend correspond to the position in Table 1.

**Figure 3.**
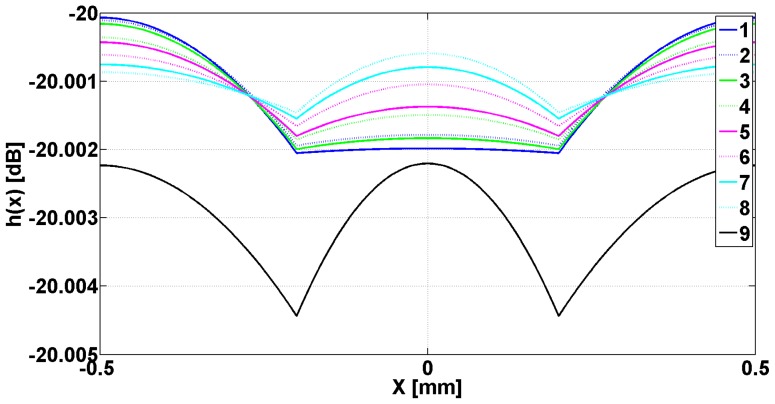
Eigenmodes *h*(*x*) plotted as a function of *x* for one period of metallic strip grating. Numbers in legend correspond to the position in Table 1.

In Figure 2 are presented the evanescent modes generated in slits when the metallic strip grating is excited with a planar TE_z_ polarized wave, and different dielectrics being inserted in slits. In Figure 3 are presented the evanescent and the abnormal modes generated in slits when the metallic strip grating is excited with a planar TM_z_ polarized wave, different dielectrics being inserted in slits. In both cases, the wavelength was 0.6 m.

Examining Figure 2, it can be observed that all the modes *e_v_*, evanescent modes have a very pronounced minimum in the middle of the slits and a maximum on the flanks of the metallic strip grating, followed by a decay toward zero in the middle of metallic strip. The evanescent and h*_v_*, abnormal modes, shown in Figure 3, have a minimum zone with almost constant amplitude in slits and maximum on the flank of metallic strips, followed by decreasing of amplitudes to middle of the metallic strips. Very closely to the interface grating — air (negative *z* near zero, Figure 1), the effect of evanescent and abnormal modes is felt, forming a near-field region. The near-field region is generally characterized as a region in space where the evanescent waves cannot be neglected and it is restricted to material boundaries; this makes evanescent wave-source decoupling impossible [24].

A solution for manipulating the evanescent and abnormal modes is to use of lenses with metamaterials [25,26]. When the effective electrical permittivity *ε_eff_*, and the effective magnetic permeability, *μ_eff_*, of a metamaterial slab are simultaneously −1, the refractive index of the slab is *n*=−*1* [27]. The surface impedance of such metamaterial is *Z* = *1*, therefore is no mismatch and consequently no reflection at the interface slab—air [26]. This metamaterial slab forms a perfect lens [25] and is focusing the electromagnetic field, and also the evanescent waves [25]. Due to experimental difficulties in obtaining a perfect lens, the manipulation of the evanescent modes can be made with this new type of electromagnetic transducer with metamaterials lens that have, at the operation frequency, either *ε_eff_* = −1 and electric evanescent modes can be manipulated, either *μ_eff_* = −1, and the lens can focus magnetic evanescent modes [28].

As shown in reference [29,30], the electric evanescent modes can be manipulated with a transducer made from a special type of metamaterial, named conical Swiss rolls, functioning in a frequency range that assures that *μ_eff_* is maximum. Working at frequencies that assure *μ_eff_* = −1, the lens with conical Swiss rolls can manipulate the magnetic evanescent modes [19,30].

**Figure 4.**
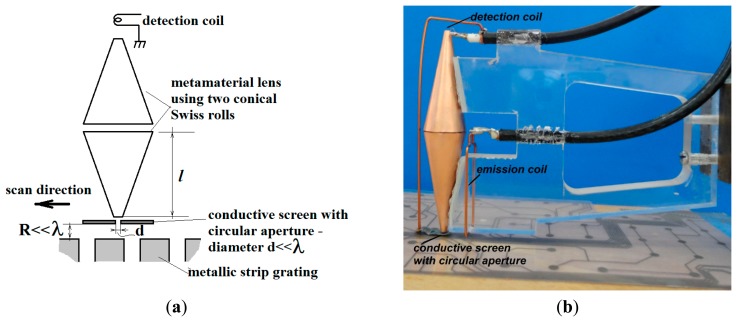
Detection of evanescent and abnormal modes generated in the slits of a metallic strip grating using lens transducer: (**a**) scheme; (**b**) photo.

The principle of the lens transducer made from two conical Swiss rolls, used for manipulating the evanescent and abnormal modes generated in slits of metallic strip grating excited with electromagnetic waves, polarized TE_z_ and respectively TM_z_ is shown in Figure 4.

The proper detection system is made from a lens transducer realized with two identical conical Swiss rolls having the large basis front to front. The focal distance of this lens is *f* ≃ *l*, where *l* represents the height of a conical Swiss roll [29]. A conductive screen with a circular aperture having the diameter *d* << *λ* (Figure 4) is placed near the focal object point. A detection coil is placed in the focal image point, converting the localized energy into an electromagnetic force (e.m.f.). The sample is raster scanned, recording the energy image pixel or electromagnetic signature.

Using the Fourier optics methods [5,31], an object *O*(*x,y*) that can represent the eigenmodes e*_v_* or h*_v_* in function of the polarization of incident electromagnetic field, has, while passing through the circular aperture and the lens, an image *I*(*x',y'*) given by:

(17) $$\begin{array}{c} I\left(x^{\prime },y^{\prime }\right)=\frac{1}{\lambda d_{1}d_{2}}\int_{-\infty }^{\infty }\int_{-\infty }^{\infty }\exp\left[i\frac{k\left({\left(x^{\prime }-x_{1}\right)}^{2}+{\left(y^{\prime }-y_{1}\right)}^{2}\right)}{2d_{2}}\right]\;P\left(x,y\right)\exp\left[i\frac{k(x_{1}^{2}+y_{1}^{2})}{2f}\right]\times \\ \left(\int_{-\infty }^{\infty }\int_{-\infty }^{\infty }O\left(x,y\right)\exp\left[i\frac{k\left({\left(x_{1}-x\right)}^{2}+{\left(y_{1}-y\right)}^{2}\right)}{2d_{1}}\right]\text{dxdy}\right)dx_{1}dy_{1} \end{array}$$

where *P*(*x,y*) is the pupil function defined as:

(18) $$p\left(x,y\right)=\{\begin{array}{cc} 1, & x^{2}+y^{2}\leq d^{2}\\ 0, & \text{otherwise} \end{array}$$

*O*(*x,y*) is the object defined as:

(19) $$O\left(x,y\right)=\{\begin{array}{llll} e_{v}\;\left(x,y\right) & \text{for} & {\text{TE}}_{\text{z}} & \text{polarized incident waves}\\ h_{v}\;\left(x,y\right) & \text{for} & {\text{TM}}_{\text{z}} & \text{polarized incident waves} \end{array}$$

*d*_1_ = *R* + *l* the distance from the object to the center of the lens
*d*_2_ = *l* the distance from the center of the lens to the detection coil.

Considering the lens transducer presented above in Figure 4, with *f* = *l*= 50 mm in front of which a conductive screen with a circular aperture having diameter d = 100 μm is placed, the effective medium of the lens presents a maximum for *μ_eff_* at the frequency of 473.8 MHz and *μ_eff_* = −1 at 476 MHz (see Section 4 for justification). The value of *R* is 75 μm, as shown in Figure 4.

Two observations must be made:

- Eigenvalues, values of β_v_, and respectively eigenmodes, *e_v_*(*x*) and *h_v_*(*x*) obtained by numerical calculation at the frequency of 500 MHz, insignificantly differ from the values obtained at frequency of 473.8 MHz for the case of TE_z_ polarization and respectively at frequency of 476 MHz for the case of TM_z_ polarization
- *e_v_* (*x*,*y*) = *e_v_* (*x*) - the electric modes have the same form and amplitude indifferent of y coordinates (Figure 1). *h_v_* (*x*,*y*) = *h_v_* (*x*)—the magnetic modes have the same form and amplitude indifferent of y coordinates (Figure 1)

**Figure 5.**
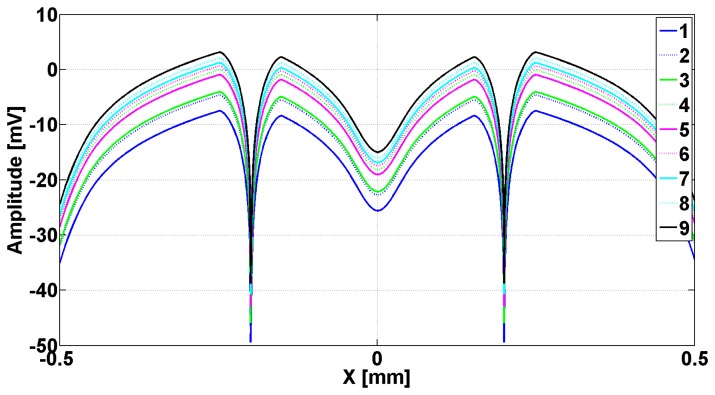
The image through transducer's lens of evanescent modes e_v_ shown in Figure 2. The numbers in the legend correspond to the position from Table 1.

Figure 5 shows the images of the evanescent modes e_v_ emphasized in Figure 2, after the passing through transducer lens, with a scanning step of 1 μm. The electromagnetic waves incident to metallic strip grating are TE_z_ polarized waves having the frequency of 473.8 MHz, which gives a maximum μ_eff_.

The analysis of curves from Figure 5 shows that the images of all e_v_ modes that are evanescent have a minimum in the middle of the slits as well as two symmetrical maxima at the distance of ±154 μm. The modes decrease toward zero on the flanks of metallic strip, and then, in the interior of the metallic strip, at the distance of ±249 μm, all the modes present other pair of maxima, followed by the decreasing of the amplitude toward the middle of metallic strips.

**Figure 6.**
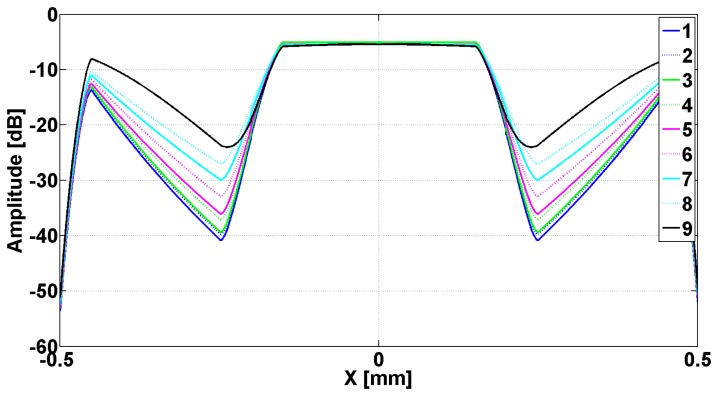
Image through transducer's lens of the evanescent modes h*_v_* presented in Figure 3. The numbers from the legend correspond to position in Table 1.

Figure 6 shows the image of the h_v_ modes, previously emphasized in Figure 3 after the passing through transducer lens. The incident waves are TM_z_ plane polarized having frequency of 476 MHz, which assures *μ_eff_* = −1.

The analysis of data from Figure 6 shows that the image of all h_v_ modes has a region of maximum in central zone of slits followed by an accentuated decreasing on metallic strip's flank that continues in the interior of the strips. This is followed by a region of increase, followed by a decrease towards zero on the middle of the metallic strip. The positions of minima as well as of maxima from the interior of metallic strips depend by the permittivity of dielectrics inserted in slits.

## 4. Samples; Experimental Set-Up

**Figure 7.**
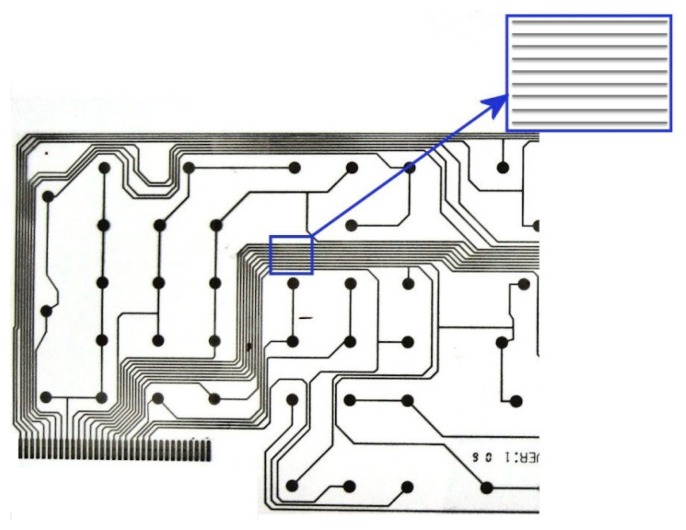
Metallic strip grating taken into study.

Metallic strip gratings having conductive traces made of silver with 10 μm thickness and 0.6 mm width deposited on polyester support with relative permittivity 4.8 have been taken into study, the distance between traces is 0.4 mm. These are portions of a flexible printed circuit board, Figure 7. At frequencies around the value of 500 MHz, the permittivity of silver is *ε_m_* = −48.8 + *j* ·3.16 [22]. The studied metallic strip grating corresponds to those used in the numerical simulations.

The transducer's lens has been realized with two conical Swiss rolls having the large basis face to face (see Figure 4). The diameter of large base is 20 mm, of small base is 3.2 mm and the height is 50 mm. The conical Swiss rolls have been made by a foil of LONGLITE™ 200, produced by Rogerscorp (Connecticut, CT, USA), having 18 μm thickness copper foil laminated adhesiveless with 12 μm thickness polyimide foil, in order to reduce the losses. Each conical Swiss roll has 1.25 turns winded on a mandrel with 20° cone angle.

The frequency dependency of lens' effective magnetic permeability has been determined measuring the *S* parameters (*S_11_* and *S_21_*) and applying the effective medium method [30,32,33]. The measurement of S parameters were made with a 4395A Network/Spectrum/Impedance Analyzer (Agilent Technologies, Santa Clara, CA, USA) coupled with *S* Parameter Test kit 87511A Agilent. The incident field is generated by one turn coil, having 16 mm average diameter from Cu wire with 1 mm diameter. The reception coil has one turn with 3 mm average diameter from Cu wire with 1 mm diameter.

**Figure 8.**
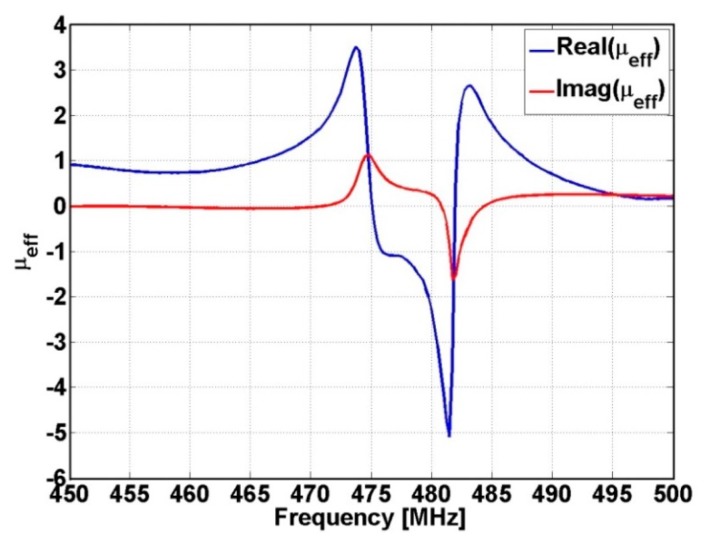
The dependence by frequency of effective magnetic permeability of the lens.

In Figure 8 is presented the dependence by frequency of effective magnetic permeability of the lens used for manipulation of evanescent and abnormal modes. It can be observed that the real component of the effective magnetic permeability reaches the maximum value at the frequency of 473.8 MHz and the value of −1 at frequency of 476 MHz, values for which the numerical simulations have been made in Section 3.2.

A conductive screen made from LONGLITE™ 200, connected to ground, having 0.1 mm diameter circular aperture, has been placed in front of the object focal point of the lens. A reception coil with one turn having average diameter of 1 mm made from Cu wire with 0.1 mm diameter, has been placed in the image focal point.

The TE_z_ polarization of the incident field was created with a rectangular frame having one turn from 1 mm diameter Cu wire. The frame having 35 × 70 mm dimensions was placed parallel with the surface of metallic strip grating at 3 mm height. The small side of the frame is placed parallel to the direction of metallic strips, the TE_z_ polarization being obtained in the central region [34]. The working frequency was 473.8 MHz.

The TM_z_ polarization was realized with the same frame, placed perpendicularly at the metallic strip grating, as it was shown in [19], the working frequency being 476 MHz. During the measurements, the excitation frame, in both configurations and the lens have been maintained in fixed position, the metallic strip grating being displaced with a motorized X-Y stage—Newmark Systems Inc. (Santa Margarita, CA, USA). The excitation frame and the reception coil are coupled with an Agilent 4395A Network/Spectrum/Impedance Analyzer. The measurement system is commanded by a PC through RS 232 for X-Y motorized stage controller and IEEE 488.2 for the Analyzer 4395A. The programs for measurements and data storage are developed in Matlab R2011b.

The electromotive force induced in the reception coil of the measurement system represents the average of 10 successive measurements in the same point, in order to reduce the effect of the white noise. The bandwidth of Analyzer 4395A was set-up at 10 Hz for diminishing the noise level.

## 5. Experimental Results; Discussions

Using the transducer lens described earlier, a region of 1 × 1 mm^2^ from metallic strip grating has been scanned with 10 μm steps in both directions. The scanning along *x* direction was made so that shall correspond to a period of the grating, *x_0_*. The same scanning parameters have been kept for the both polarizations of the incident electromagnetic field and for the two operation frequencies.

**Figure 9.**
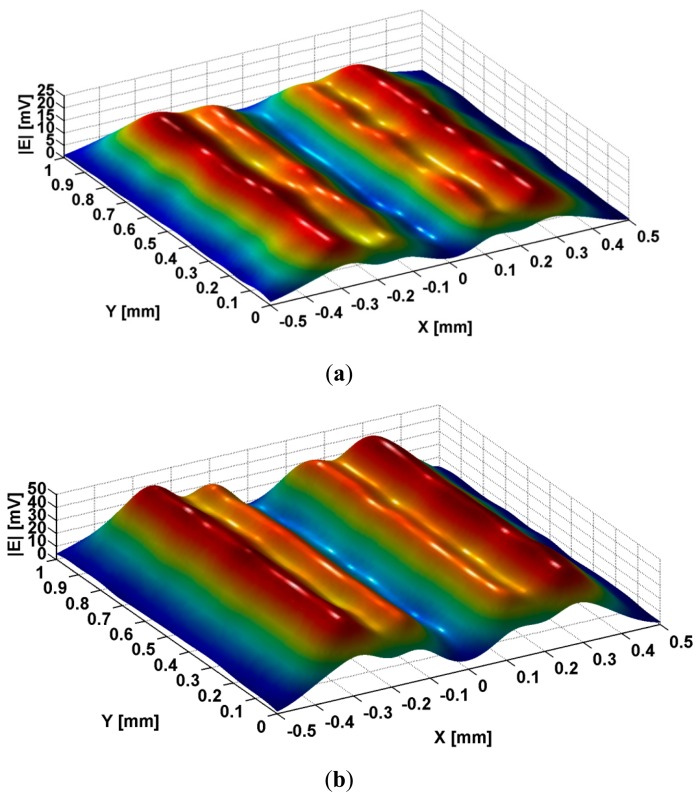
The image of the evanescent modes generated in slits for TE_z_ polarized excitation at 473.8 MHz frequency: (**a**) slits filled with air; (**b**) slits filled with isopropyl alcohol.

In Figure 9a is presented the image of the electric evanescent mode generated in slits, in air, when metallic strip grating is excited with a TE_z_ polarized wave at frequency of 473.8 MHz. When the slits are filled with air, the profile of evanescent waves e_v_ generated in slits at TE_z_ polarized wave excitation is kept. Along *y* coordinate, the amplitude of the signal induced in the reception coil remains approximately constant, confirming the theoretical considerations *e_v_* (*x*, *y*) = *e_v_* (*x*). Along *x* coordinate, for *x_0_* period, are observed the same minima of the evanescent modes e_v_, in the middle of the slits, the symmetrical maxima at ±155 μm, with decreasing towards the minimum value, but different by zero, on the strips flanks. In the interior of the strips, it can be observed other pair of maxima, at ±270 μm, followed by the decreasing towards zero on the middle of the strip. It can be noticed that the visualization of these modes can be improved using metamaterials lens in order to manipulate the evanescent waves that appear in slits. If the slits are filled with isopropyl alcohol, the image of the evanescent mode is modified, as can be seen in Figure 9b.

It can be observed that the amplitude of the signal induced in the reception coil increases when a dielectric with high dielectric constant (isopropyl alcohol) is inserted in slits, fact that confirms the theoretical predictions presented in Figure 5. On the middle of slit (*x* = 0), as well as on the middle of the metallic strip (*x* = ±0.5 mm), the signal has minimum approximately equal to zero. Four maxima of e.m.f. induced in the reception coil appear, two located at the interface metallic strip—slits and other two located in the interior of metallic strip on the one side and on the other side of the slit. The images present an increasing of the amplitude of the signal induced in the reception coil confirming the theoretical estimation of liquid dielectric effects over the evanescent waves from slits and constitutive parameters. The approach is original because emphasizes the propagation of evanescent waves through dielectric fluids that fill the slits.

**Figure 10.**
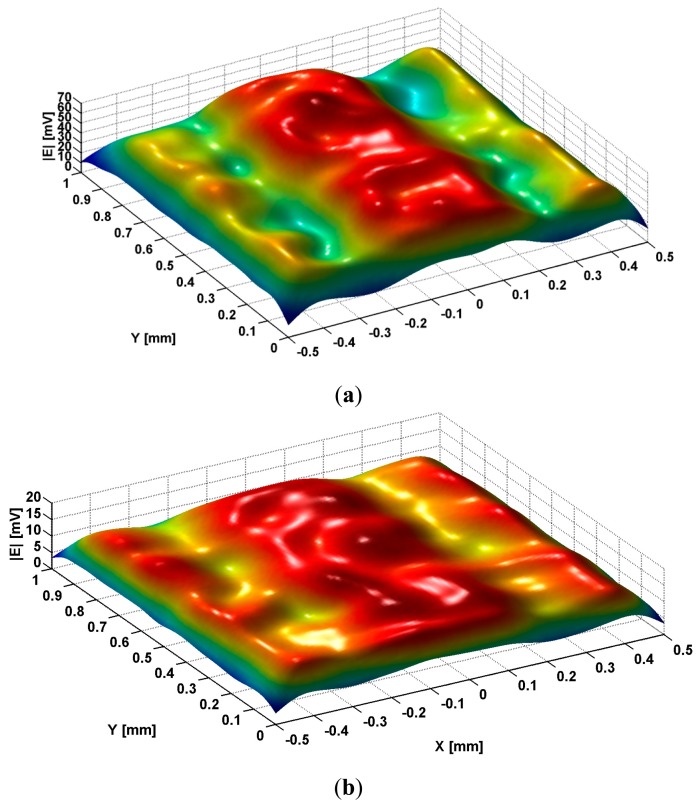
Image of evanescent and abnormal modes generated in slits for TM_z_ polarized excitation at frequency of 476 MHz: (**a**) slits filled with air; (**b**) slits filled with isopropyl alcohol.

When a TM_z_ polarized field having the frequency of 476 MHz acts over the metallic strip grating, scanning the same region of 1 × 1 mm^2^ with 10 μm step on both directions, the detection being made with the same type of electromagnetic transducer, the image of the evanescent and abnormal modes created in slits is presented in Figure 10.

When the slits are filled with air, according to Table 1, only one evanescent mode will be generated, the amplitude of the signal induced in the reception coil having the shape presented in Figure 10a. The amplitude presents a region of maximum in the central zone of the slit followed by an accentuated decreasing towards the flanks of metallic strips. In the metallic strips there are two secondary maxima, with amplitude smaller than the one in the central zone of the slit followed by a decreasing to zero to the middle of metallic strip. The existence of a single evanescent mode, theoretical foreseen in Figure 6, is experimentally confirmed by the existence of a local maximum in the middle zone of the slits, with maximum amplitude on the middle of the slits, followed by an accentuated decreasing, symmetrically on the flanks of the strips. In strips, at the distance of ±42 μm from the vertical wall of the strip, localized at ±0.2 mm, there are two secondary maxima with amplitudes smaller than those in the central zones with approximately 0.82 times. These results are in good accordance with theoretical estimations.

It can be observed that in the case of excitation with TM_z_ polarized wave, for large values of the liquid dielectric constants larger than 10, according to Table 1, when ε_d_ = 17.9, (isopropyl alcohol ), in slits are generated abnormal modes.

Because the real component of the propagation constant β_v_ for isopropyl alcohol is smaller than the imaginary component, abnormal modes will be generated in slits, the electromagnetic image of these modes shows a similar behavior like in the case of air in the slits. The amplitude of the signal is smaller and has a central maximum more flat (Figure 10b).

For the case of modes excited by TM_z_ polarized electromagnetic waves, their shape detected with transducer lens is also corresponding with those theoretically foreseen and presented in Figure 6. In the case in which the scanning step is increased at 0.1 mm, the shape of the mode presented in Figure 10a is close to the one previous presented [19].

This opens a large perspective for the use of transducer with metamaterials lens in sub-subwavelength regime as sensors (including biosensors based on the evanescent modes generated in slits and extremely low frequency plasmons). In the same time, due to the carbon fibers conductivity, the CFRP can be seen as stratified conductive strip gratings structure. Nowadays, CFRP are used in different domains, especially in aeronautics. This is justifying the development of new noninvasive electromagnetic testing methods with better sensitivity, in order to detect and evaluate delaminations, moisture presence, fibers breaking, *etc.*

## 6. Conclusions

The current paper proposes to study the eigenmodes that appear in metallic strip gratings made of silver, when both TE_z_ and TM_z_ incident waves are considered in sub-subwavelength regime. The TE_z_ and TM_z_ polarization of incident field have been created with a rectangular frame with the plane parallel for TE_z_ and respectively perpendicular for TM_z_ to metallic strip surface and it was fed with alternative current.

An analytic model was developed in order to calculate the eigenmodes and respectively eigenvalue in a thick conductive strip grating, which shows that in a metallic strip grating having silver strip with geometrical dimension x_m_ = 0.6 mm, x_d_ = 0.4 mm, h = 10 μm, excited with a TM_z_ and TE_z_ polarized electromagnetic wave with 500 MHz frequency, abnormal and/or evanescent modes appear. It must be mentioned that the values obtained by numerical calculus at 500 MHz frequency, no significant differ from those obtained at 473.8 MHz for TE_z_ polarization and 476 MHz for TM_z_ polarization.

In order to detect and intensify of the abnormal and/or evanescent modes, a metamaterial lens has been developed using two conical Swiss rolls. The study focuses on the appearance of abnormal and/or evanescent modes for the cases where various dielectric fluids fill the gaps between the strips, from both an experimental and theoretical points of view. The experimental study confirms the theoretical findings according to which, when a TE_z_ polarized wave is used, the amplitude of the signal induced in the reception coils is modified when a dielectric fluid with a high dielectric constant is used. When a TM_z_ polarized wave is used, the existence of abnormal modes is experimentally confirmed, if the space between the metallic strip gratings is filled with a dielectric.

Using the transducer and the procedure mentioned earlier, interruptions, short circuits of metallic strips of printed circuits boards as well as non-alignment of carbon fibers, lack of resin or voids, and delamination induced by low-energy impacts can be detected.

## Figures and Tables

**Table 1.** Eigenvalues for Equations (8) and (9) for slits filled with liquid dielectrics.

No.	Dielectric in Slits	Dielectric Constant ε_d_	*β_v_*		Observations
			TE_z_ Excitation	TM_z_ Excitation	
1	Air	1	0 + i·10.470	0 + i·16.875	For both excitations in slits, only one evanescent mode appears

2	Carbon tetrachloride	2.24	0 + i·15.670	0 + i·25.870	For both excitations in slits, only one evanescent mode appears

3	Benzene	2.27	0 + i·15.780	0 + i·25.900	For both excitations in slits, only one evanescent mode appears

4	Chloroform	4.81	0 + i·22.970	0.2 + i·39.000	For TE_z_ polarization in slits only one evanescent mode appears
					For TM_z_ polarization there it is an abnormal mode

5	Chlorobenzene	5.62	0 + i·24.830	0.3 + i·43.100	For TE_z_ polarization in slits only one evanescent mode appears
					For TM_z_ polarization there it is an abnormal mode

6	Tetrahydrofuran	7.58	0 + i·28.831	0.5 + i·52.100	For TE_z_ polarization in slits only one evanescent mode appears
					For TM_z_ polarization there it is an abnormal mode

7	Dichloromethane	8.93	0 + i·31.290	0.7 + i·58.035	For TE_z_ polarization in slits only one evanescent mode appears
					For TM_z_ polarization there it is an abnormal mode

8	*o*–Dichlorobenzene	9.93	0 + i·33.073	0.9 + i·62.511	For TE_z_ polarization in slits only one evanescent mode appears
					For TM_z_ polarization there it is an abnormal mode

9	Isopropyl alcohol	17.9	0 + i·44.342	4.2 + i·104.321	For TE_z_ polarization in slits only one evanescent mode appears
					For TM_z_ polarization there it is an abnormal mode
